# Second Advent of Thomsonism

**Published:** 1848-02

**Authors:** 


14. *Second Advent of Thomsonism.*—That nothing may be wanting, in the metropolis of New England, to meet all the whims and caprices of all classes of society, the whole Botanico-Medical College of Ohio (supposed to be Thomsonian) —that is, its faculty, which embraces the lion of the party, the chancellor of the University himself—have been transferred to Bromfield street, Boston, within a stone's throw of the Medical Journal office. Five lectures are to be given daily. What is to become of that monster of a Cayenne College at Worcester, Mass., which was to swallow up all the venerated institutions of medicine in Massachusetts?—It is evident that the two will soon be at loggerheads, since the Worcester gentlemen, who predicted the infliction of a Thomsonian governor over the Commonwealth, in 1849, in case a charter was refused them, will logically demonstrate that these Buck-eye botanics are trenching on their own hallowed ground. For ourselves, not the least fear is entertained of the efforts of these loud-talking strangers. It was benevolent in them to commence operations in Boston, the focus of Thomsonian ignoramuses. If it is possible to enlighten the latter, the attempt should be made, as they actually disgrace their calling. It is generally supposed that all who practise, as these New England one-idea lobelia people do, are equally stupid; and if measures are in progress for letting light in upon their No. 6 minds, all well wishers to humanity will hail the intelligence with delight. After hearing some of the lecturers from Ohio, a further notice may be given of their character and tendency.—*Boston Medical and Surgical Journal.*

